# The Growing Role of Intracardiac Echo in Congenital Heart Disease Interventions

**DOI:** 10.3390/jcm14072414

**Published:** 2025-04-01

**Authors:** Eihab Ghantous, Jamil A. Aboulhosn

**Affiliations:** Ahmanson/UCLA Adult Congenital Heart Disease Center, Los Angeles, CA 90095, USA; jaboulhosn@mednet.ucla.edu

**Keywords:** intracardiac echo, Adult Congenital Heart Disease, transcatheter pulmonary valve replacement, atrial septal defect, ventricular septal defect

## Abstract

Advancements in congenital heart disease (CHD) care have significantly improved survival, leading to a growing population of adults with congenital heart disease (ACHDs). Many of these patients require ongoing interventions for residual defects, conduit or valve dysfunction, and arrhythmia management, often performed using transcatheter techniques. Imaging plays a critical role in ensuring procedural success and safety. Intracardiac echocardiography (ICE) has emerged as an essential imaging modality in ACHD interventions. With continuous technological advancements, ICE offers several advantages over transesophageal echocardiography (TEE) and transthoracic echocardiography (TTE), including superior visualization, real-time guidance, and the ability to avoid general anesthesia. These benefits have made ICE the preferred imaging tool for many transcatheter procedures. This review explores the expanding role of ICE in ACHD interventions, highlighting its applications in structural and electrophysiological procedures. By enhancing procedural precision and reducing complications, ICE is transforming the management of ACHD patients, optimizing outcomes, and improving long-term care for this complex and growing population.

## 1. Introduction

The incidence of congenital heart disease (CHD) varies from about 4 to 50 per 1000 live births [1]. Advancements in the congenital heart disease diagnosis, treatment and interventions have significantly reduced mortality rated and improved quality of life for millions of patients [2,3]. As a result, the population of adults with congenital heart disease (ACHDs) exceeds that of pediatric patients with congenital heart disease [4,5]. These individuals often require multiple interventions throughout their lives, many of which can be performed using a transcatheter approach in the catheterization laboratory. Selecting the appropriate imaging technique for each procedure is critical to ensure procedural success.

Meanwhile, intracardiac echocardiography (ICE), first introduced in the 1960s [6], has evolved and become an integral part of different electrophysiology and percutaneous interventional procedures in adult cardiology. ICE offers distinct advantages over transesophageal echocardiography (TEE), including its ability to be performed without the need for endotracheal or endoscopic intubation, eliminating the risk of esophageal trauma and the need for an additional operator, as well as the ability to perform it without the need for sedation in patients. Moreover, ICE is feasible in patients with contraindications for TEE such as those with esophageal strictures or recent esophageal surgery. One population that may have great benefit from ICE are patients with cirrhosis who may have esophageal varices as well as slow hepatic elimination of sedation. Additionally, ICE use has been associated with a reduction in radiation exposure during procedures [7,8].

ICE can be interchangeable with TEE for certain interventions such as ostium secundum atrial septal defect closure and patent foramen ovale closure. However, in our experience, ICE offers distinct advantages over TEE, particularly in imaging right-sided structures and guiding right-sided valvular interventions. The pulmonary valve, often poorly visualized with TEE, can be clearly assessed using ICE from within the right ventricle. Additionally, ICE provides excellent visualization of the tricuspid valve, facilitating precise guidance during interventions. This modality effectively overcomes the far-field imaging limitations of TEE and mitigates shadowing artifacts caused by prosthetic materials or septal patches.

However, the use of ICE is not without its challenges. It carries an increased risk of bleeding due to vascular injury, as venous access is required. This risk can be mitigated with operator experience and proper technique. Some of the available ICE systems and differences have been described in previous publications [8,9]. Furthermore, recent advancements led to the approval of new ICE catheters by the FDA, offering enhanced image quality and advanced features such as three-dimensional (3D) and four-dimensional (4D) imaging.

This review highlights the application of ICE in select ACHD procedures, emphasizing its growing role in optimizing outcomes for this unique patient population.

## 2. Acquiring Baseline Images

ICE catheters require a minimum 6 French (Fr) venous sheath [10]; however, larger sheaths are necessary for 3D ICE catheters, such as a 10 Fr sheath for the VeriSight ICE catheter (Philips) [11] and a 14 Fr sheath for the Acuson ICE catheter (Siemens) [12]—two of the most widely used 3D catheters in the U.S. Advancing the catheter through the femoral vein into the right atrium (RA) can be performed without fluoroscopy. The key principle is to keep the vein, mainly the inferior vena cava (IVC), which appears as a black space, in front of the catheter and avoid advancing when an echogenic (white) structure obstructs the path. If an angle appears, gentle retroflexion or anteflexion maneuvers help maintain the vein view.

**Figure 1:**
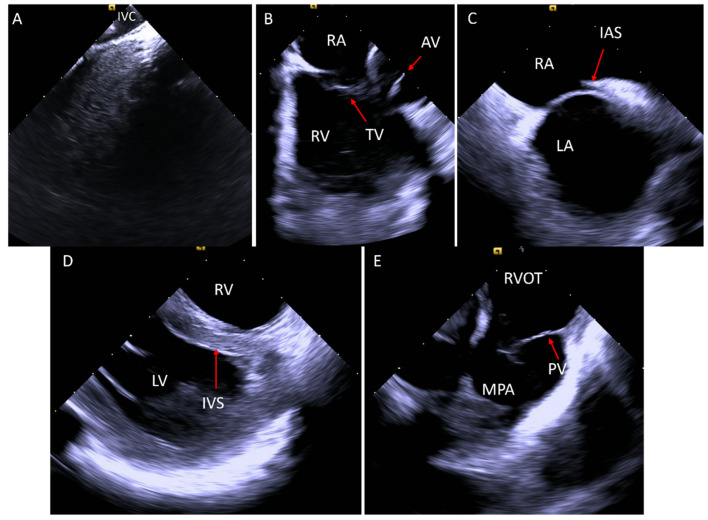
(**A**) ICE catheter in the IVC showing the IVC cavity in black. (**B**) The “home view”: ICE catheter in the RA looking at the TV and the RV. (**C**) After clockwise rotation of the ICE catheter, the IAS appears. (**D**) While in the RV, the LV is better visualized with the IVS. (**E**) After clockwise rotation of the catheter from the RV, the RVOT is seen with the PV and the MPA. AV, aortic valve; IAS, interatrial septum; ICE, intracardiac echo; IVS, interventricular septum, LA, left atrium; LV, left ventricle; MPA, main pulmonary artery; PV, pulmonary valve; RA, right atrium; RV, right ventricle; RVOT, right ventricular outflow tract.

Catheter advancement continues until the “home view” is achieved, where the catheter is positioned in the RA, visualizing the tricuspid valve (TV) with a clear view of the right ventricle (RV). Rotating the catheter clockwise from this position sequentially reveals the aortic valve, right ventricular outflow tract (RVOT), portions of the left ventricle (LV), mitral valve (MV), interatrial septum, and left atrial appendage (LAA). For optimal visualization of the pulmonary valve (PV) and distal RVOT structures, the catheter is advanced to the RV. This is achieved by directing it inferiorly from the “home view” while keeping part of the TV in sight and then gently advancing into the RV. Once in the RV, further clockwise rotation sequentially displays the interventricular septum, RVOT, and PV (Figure 1). These baseline imaging techniques, with adjustments based on individual anatomy, provide the necessary visualization for procedural guidance.

## 3. Ostium Secundum Atrial Septal Defect (ASD) and Patent Foramen Ovale (PFO) Closure

Atrial septal defects are among the most common congenital heart defects, accounting for approximately 10–15% of such cases [13,14]. Closure of an ostium secundum ASD is recommended as a transcatheter procedure when feasible, according to both the European and American guidelines [15,16]. Historically, these procedures were performed under the guidance of TEE. However, with the advancement of ICE, it now competes with TEE as the preferred imaging modality for guiding these interventions [17,18,19].

**Figure 2:**
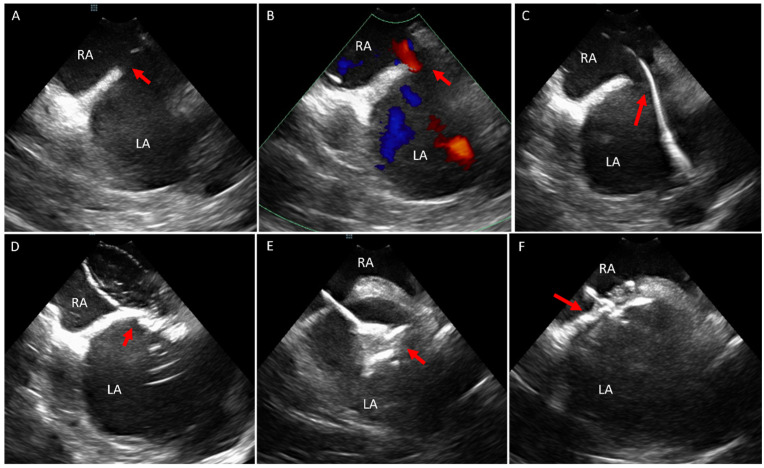
(**A**,**B**) ICE catheter positioned in the right atrium looking at the interatrial septum and visualizing the ostium secundum ASD (red arrow) without color Doppler (**A**) and with color Doppler (**B**) showing left to right shunt. (**C**) Wire (red arrow) across the ostium secundum ASD towards the left upper pulmonary vein. (**D**) Balloon occlusion of the defect (red arrow). (**E**) Deployment of the left atrial disc of the atrial septal defect occluder device (red arrow). (**F**) Deployment of the right atrial disc of the atrial septal occlude device (red arrow) and showing a good alignment of the device with the atrial septum. ICE, intracardiac echo; LA, left atrium; RA, right atrium.

**Figure 3:**
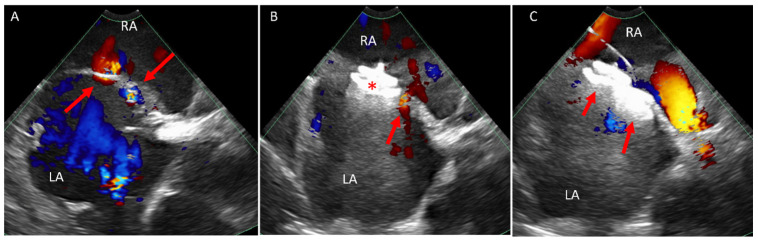
(**A**) Color Doppler with the ICE catheter in the right atrium showing evidence of two atrial septal defects (red arrows). (**B**) Color Doppler image post deployment of the first atrial septal defect device occluder (asterisk) with residual left to right shunt (red arrow). (**C**) Color Doppler showing two ASD occluder devices deployed (red arrows) with no residual shunt. ASD, atrial septal defect; ICE, intracardiac echo; LA, left atrium; RA, right atrium.

The standard imaging views and the procedural protocols for ICE-guided ASD closure have been described in previous literature [8,20]. In our practice, we begin by confirming the presence of the defect using two-dimensional (2D) imaging and color Doppler to exclude other defects within the interatrial septum. ICE is then used to guide critical procedural steps, including positioning of the wire in the pulmonary vein (typically the left upper pulmonary vein), performing balloon sizing and stop-flow assessment, and deploying the closure device (Figure 2). At the end of the procedure, ICE is utilized to confirm the absence of complications such as device embolization, pericardial effusion or valvular injury. Advances in imaging technology have also facilitated the management of more complex cases, such as the closure of multiple ASDs using ICE guidance alone (Figure 3).

**Figure 4:**
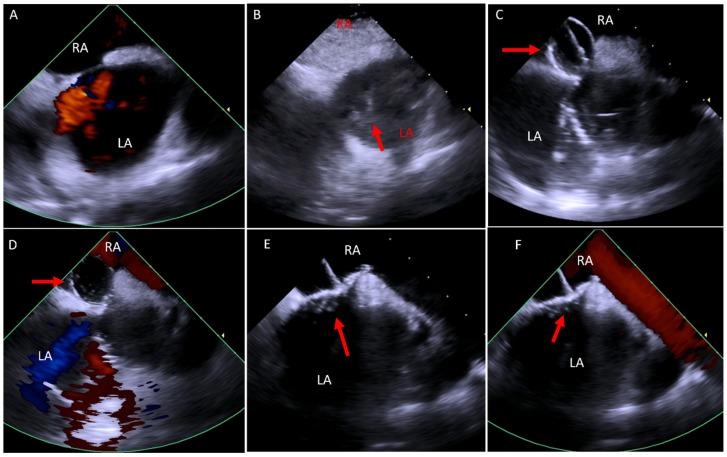
ICE catheter in the right atrium. (**A**) Color Doppler on the interatrial septum showing no evidence of shunt. (**B**) Agitated saline injection in the femoral vein with Valsalva maneuver showing positive study with bubbles crossing to the left atrium (red arrow) and confirming the diagnosis of PFO. (**C**,**D**) Balloon sizing and occlusion of the PFO without (**C**) and with (**D**) color Doppler, showing no evidence of further defects in the septum. (**E**,**F**) Deployment of PFO occluder (red arrow) without (**E**) and with (**F**) color Doppler and showing no residual shunt. ICE, intracardiac echo; LA, left atrium; PFO, patent foramen ovale; RA, right atrium.

For PFO closure, ICE has become the preferred method of imaging during the procedure in the vast majority of cases. The use of ICE for procedural guidance closely mirrors its application in ostium secundum ASD closure. An example of this procedure is illustrated in Figure 4.

## 4. Ventricular Septal Defect (VSD)

**Figure 5:**
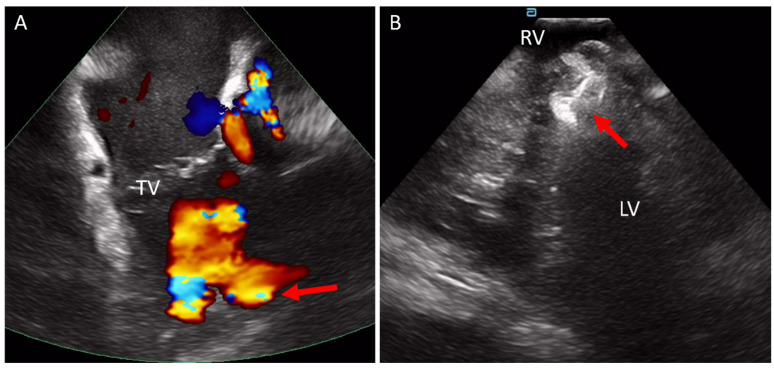
ICE catheter in the right atrium. (**A**) Color Doppler from the right atrium, looking through the tricuspid valve (TV) at the right ventricle and visualizing color flow through the muscular septum consistent with a muscular VSD (arrow). (**B**) ICE catheter within the RV. The muscular VSD occlude seen after deployment. ICE, intracardiac echo; LV, left ventricle; TV, tricuspid valve; VSD, ventricular septal defect.

Percutaneous closure of VSDs is feasible for muscular or apical VSDs and select membranous VSDs [21,22]. The guidelines recommend VSD closure when there is evidence of LV volume overload, history of endocarditis, especially if recurrent, progressive aortic regurgitation from prolapse of the aortic valve cusp due to the VSD, significant left to right shunt without significant pulmonary vascular resistance [15,16]. Traditionally, this procedure is performed under the guidance of TEE or transthoracic echocardiography (TTE). However, since the introduction of ICE, it has also been employed in this context. A study evaluating 12 cases of percutaneous membranous VSD closure with both ICE and TEE found that ICE provided comparable measurement of the defect to TEE. The study concluded that ICE has the potential to replace TEE in most of these cases [23]. ICE offers unique advantages, including its proximity to the defect and ability to avoid shadowing caused by prosthetic material or the lungs (Figure 5).

## 5. Valvular Interventions

The use of ICE is well-established in transcatheter aortic valve replacement (TAVR) procedures [24,25,26] and its application is increasing in transcatheter edge-to-edge repair of the mitral valve [27]. These procedures are predominantly performed by a non-ACHD structural interventionalist. This review will focus on interventions involving right-sided valves.

### 5.1. Paravalve Leak (PVL) Occlusion

**Figure 6:**
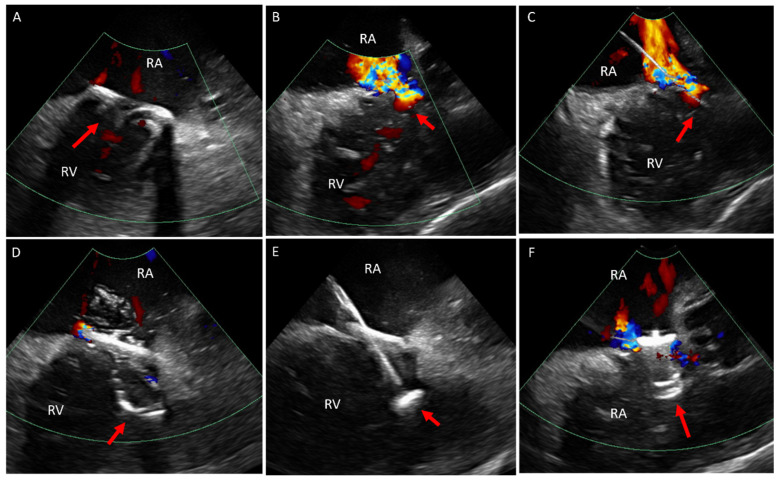
ICE catheter within the right atrium. (**A**) Color Doppler at the tricuspid valve (arrow) showing no intravalvular regurgitation. (**B**) Color Doppler when rotating the ICE catheter medially shows severe medial paravalve regurgitation (arrow). (**C**) Color Doppler at the tricuspid valve annulus showing a wire crossing the paravalve area. (**D**) Color Doppler while balloon (arrow) sizing and occluding the paravalve leak showing only minimal residual leak. (**E**) Showing the closure device attached to a cable across the defect and in the RV. (**F**) Color Doppler after deployment and release of the closure device in the paravalve leak area showing only mild residual leak. ICE, intracardiac echo; RA, right atrium; RV, right ventricle.

In the ACHD population, surgical valvular replacement is a common intervention. While these procedures significantly enhance patient longevity and quality of life, they are not without complications. One such complication is PVL, which has been reported in up to 18% of cases involving the aortic position and 23% involving the mitral position [28,29]. Percutaneous PVL occlusion has emerged as an effective and less invasive alternative to surgery [30,31]. While these procedures are traditionally performed under TEE guidance, our experience has shown that ICE is particularly valuable, especially for PVL involving the right atrioventricular valve (Figure 6) or the pulmonary valve.

### 5.2. Transcatheter Tricuspid (Right Atrioventricular) Valve in Valve or Valve in Ring Replacement (TVIV/TVIR)

Tricuspid valve regurgitation (TR) significantly impairs quality of life and is associated with increased mortality [32]. Although TVIV for a native tricuspid valve has only recently been introduced as a treatment option for severe TR [33], prior surgical interventions involving the tricuspid valve are common in the ACHD population, such as patients with Ebstein’s Anomaly and Tetralogy of Fallot patients with secondary TR. Once a valve or annuloplasty ring has been implanted in the tricuspid position, TVIV/TVIR becomes a viable alternative to high-risk surgical intervention [34].

**Figure 7:**
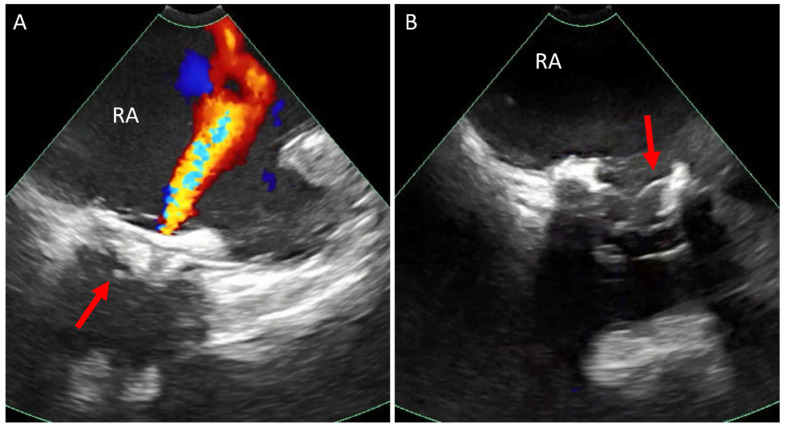
ICE catheter in the right atrium. (**A**) Color Doppler at the tricuspid valve in a 26-year-old patient with severe prosthetic tricuspid valve stenosis, showing calcified valve (arrow) with at least moderate regurgitation. (**B**) Color Doppler view in systole post TVIV showing no regurgitation and the new valve leaflets are well seen (arrow). ICE, intracardiac echo; RA, right atrium; TVIV, transcatheter tricuspid valve-in-valve replacement.

Due to the anterior position of the tricuspid valve, TEE provides suboptimal visualization and procedural guidance. Consequently, these procedures rely on a combination of fluoroscopy, TTE, and ICE [34]. In our experience, ICE provides superior visualization of the anatomy both before and after valve implantation, and is utilized to look for procedural complications (Figure 7).

### 5.3. Transcatheter Pulmonary Valve Replacement (TCPVR)

Many ACHD patients have undergone surgeries involving the right ventricular outflow tract (RVOT) often utilizing conduits and valves [2,3]. These include patients with Tetralogy of Fallot, congenital aortic stenosis patients post Ross procedure, patients post Rastelli procedure and others. Over time, these conduits and valves are prone to dysfunction, necessitating further intervention. Transcatheter pulmonary valve replacement (TCPVR) was introduced in 2000 [35] and nowadays has become the most commonly performed transcatheter valve procedure in ACHD patients [36,37,38,39].

**Figure 8:**
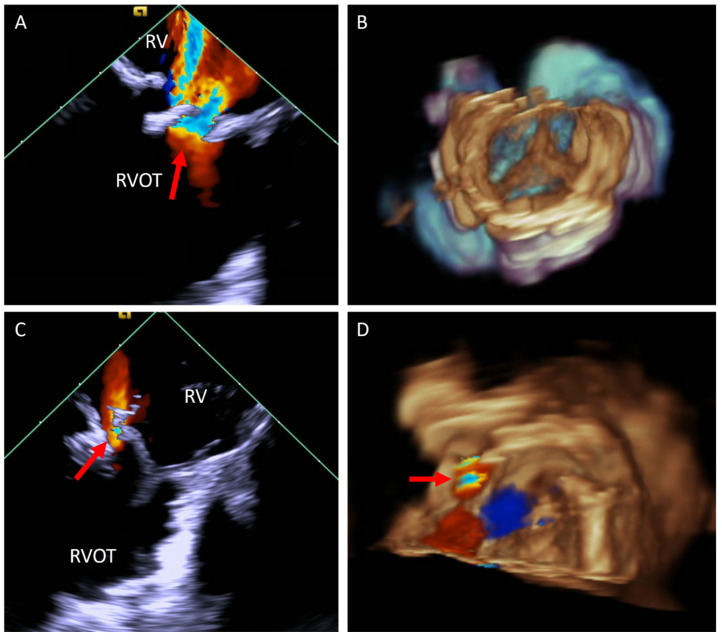
ICE catheter in the right ventricle. (**A**) Color Doppler image at the RVOT showing severe pulmonary regurgitation (arrow). (**B**) Three-dimensional image of a valve post TCPVR showing the three valve leaflets. (**C**,**D**) Color Doppler of the RVOT in 2D (**C**) and 3D (**D**) post TCPVR showing mild paravalve leak (arrow) with the 3D image showing the exact location of the paravalve leak. ICE, intracardiac echo; RVOT, right ventricular outflow tract; TCPVR, transcatheter pulmonary valve replacement.

Due to the limitation of TTE and TEE in visualizing the RVOT conduit and pulmonary valve, these procedures have historically relied mainly on fluoroscopy. However, advancements in ICE technology have expanded its application, offering improved visualization and procedural guidance [37,40,41]. In our practice, we use 4D ICE at baseline to confirm the diagnosis and assess for additional defects as well as post valve implantation to evaluate valve function and procedural complications (Figure 8).

## 6. ICE in Specific Congenital Cases

### 6.1. Baffle Leak Occlusion

Older patients with dextrotransposition of the great arteries who underwent Mustard or Senning atrial switch operations [42,43] are prone to complications, including stenosis or obstruction of the systemic or pulmonary venous baffle [44,45]. Additionally, baffle leaks are observed in up to 65% of these patients [46]. While most of these procedures are carried out using fluoroscopy alone or with TEE, multiple case reports have demonstrated the feasibility of using ICE in these procedures [47,48,49]. The procedural steps are similar to those for ostium secundum ASD closure, as previously described.

### 6.2. Fontan Fenestration Occlusion

Patients with univentricular heart physiology often survive into adulthood after multiple surgical interventions, with Fontan circulation serving as the final stage of single-ventricle palliation. In some cases, a fenestrated Fontan is created, and while the decision to make or close the fenestration depends on multiple clinical, imaging, and hemodynamic considerations beyond the scope of this paper, transcatheter closure is possible using covered stents or occlusion devices [50]. Most of these procedures are performed utilizing TEE, but recent reports suggest that ICE is a feasible imaging alternative in these cases [51].

## 7. ICE as a Diagnostic Tool

**Figure 9:**
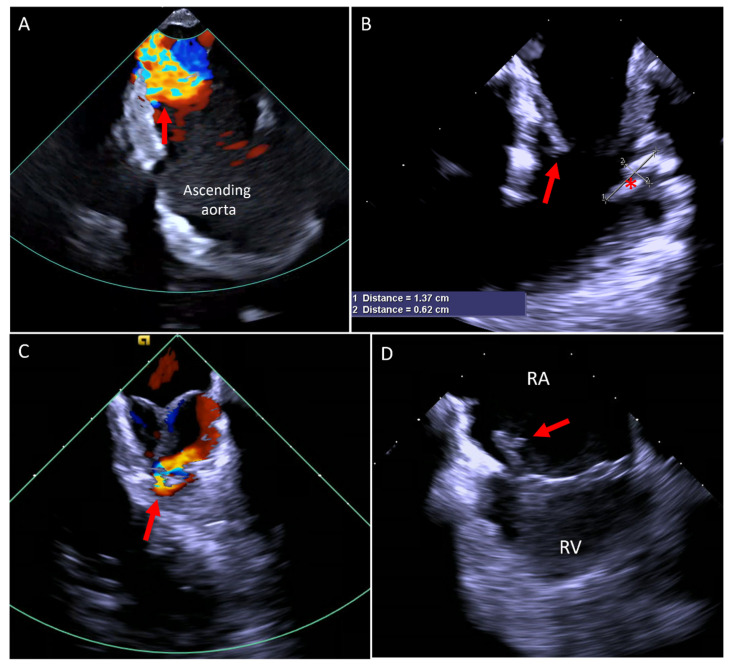
(**A**) Color Doppler image taken from the RVOT area, looking at the aortic valve showing severe aortic regurgitation (arrow). (**B**) RVOT imaging showing a thrombus (asterisk) distal to the pulmonary valve (arrow). (**C**) Color Doppler at the RVOT showing aortopulmonary fistula (arrow). (**D**) Two-dimensional image from the RA showing a thrombus on the pacemaker lead (arrow). RA, right atrium; RV, right ventricle; RVOT, right ventricular outflow tract.

Due to its close proximity to cardiac structures, ICE overcomes many of the imaging limitations associated with other modalities, such as TTE, TEE, or MRI, which are affected by shadowing from the lungs, ribs, or prosthetic materials. In our experience, ICE has proven invaluable in diagnosing previously undetected cardiac conditions, as illustrated in Figure 9.

## 8. ICE in the Electrophysiology (EP) Procedures

ICE is widely used in EP procedures, starting with transeptal puncture [52] for left heart access, detailed visualization of the pulmonary veins, the proximity of the valves and the esophagus in relation to the pulmonary veins, while also enabling the identification of thrombi in the left atrial appendage and monitoring for early complications, especially in the atrial fibrillation ablation and left atrial appendage occlusion procedures [53,54,55]. Furthermore, ablation of anatomical sources of atrial fibrillation beyond the pulmonary veins has been explored; however, it carries an increased risk of damaging adjacent structures, such as the phrenic nerve and sinus node. Recently, ICE-guided pulsed field ablation has demonstrated the ability to effectively and safely isolate the superior vena cava as a source of atrial fibrillation [56].

**Figure 10:**
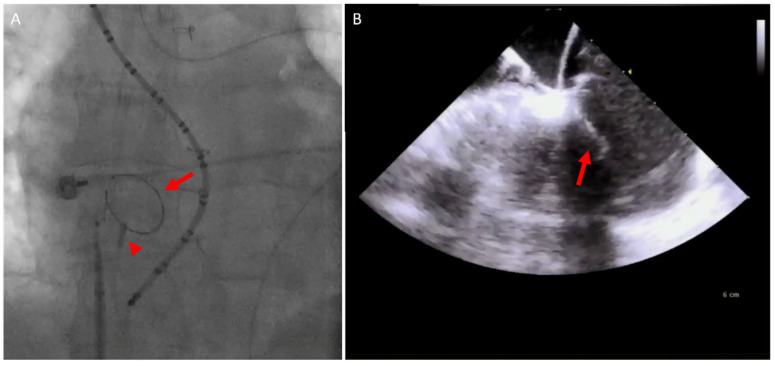
Simultaneous fluoroscopy and ICE imaging of a D-TGA patient post Mustard operation during the baffle puncture in an atrial flutter ablation procedure. (**A**) Fluoroscopy showing the ICE catheter (arrowhead) and the wire inside the pulmonary venous atrium (arrow). (**B**) ICE image showing the wire crossing the baffle to the pulmonary venous atrium (arrow). D-TGA, dextrotransposition of the great arteries; ICE, intracardiac echo.

In the congenital patients, ICE adds further value by facilitating baffle or conduit punctures, enhancing procedural safety without requiring additional operator (Figure 10) [57].

## 9. ICE Application in Interventional Cardiology in the Non-Congenital World

ICE has been widely utilized in interventional cardiology across various procedures. In transcatheter aortic valve replacement (TAVR), ICE offers several advantages, including the ability to perform the procedure without requiring patient intubation, continuous real-time monitoring, the absence of fluoroscopic interference, and precise Doppler-based assessment of pulmonary artery pressures. Additionally, its use has been associated with a reduced risk of permanent pacemaker implantation following TAVR with a self-expandable valve [25,26]. ICE has also been found to be a feasible and safe imaging modality for transcatheter edge-to-edge mitral valve repair, requiring only conscious sedation [27]. A similar approach has proven effective for transcatheter edge-to-edge repair of the tricuspid valve as well [58].

## 10. The Cost Effectiveness of ICE

As discussed earlier, ICE offers multiple advantages, though it may incur a higher upfront cost in certain procedures. For instance, in atrial fibrillation ablation, ICE has been associated with lower in-hospital mortality, fewer procedural complications, and shorter length of stay, yet it also leads to increased hospitalization costs [59]. Similarly, in left atrial appendage closure (LAAC), ICE has been estimated to increase costs by approximately USD 1700 compared to TEE [60], though some reports suggest comparable overall expenses [61]. However, financial considerations vary over time and across different healthcare systems, as costs are influenced by institutional pricing, reimbursement policies, and evolving technology.

Despite these financial factors, ICE provides significant clinical advantages in select patient populations. It is particularly beneficial for patients with esophageal pathology, those who prefer to avoid deep sedation or general anesthesia, or those at higher risk for anesthesia-related complications (e.g., patients with advanced parenchymal lung disease). Additionally, ICE is especially useful for guiding right-sided heart interventions, such as tricuspid or pulmonary valve procedures, where synthetic materials may interfere with TEE imaging. While ICE may result in higher hospital charges, TEE generally incurs greater professional fees and requires an additional operator, impacting overall procedural efficiency and resource utilization [61].

## 11. Future Perspectives

ICE has the potential to serve as the sole imaging modality in a wide range of structural and electrophysiology (EP) procedures, including in the ACHD world. However, its widespread adoption is currently hindered by several limitations, including the high cost of existing catheters, restricted spatial resolution, limited 3D volumetric imaging capabilities, and the requirement for larger sheaths to accommodate higher-quality ICE catheters. These challenges are actively being addressed, and advancements in technology are expected to yield next-generation ICE catheters that are smaller in size and capable of delivering superior spatial resolution and enhanced 3D imaging. Additionally, the future of ICE may see the integration of artificial intelligence (AI) and deep learning models into imaging systems with the potential of automated image optimization by adjusting gain, depth, and focus dynamically, ensuring the best visualization without manual adjustments. Furthermore, machine learning models can predict the safest and most efficient catheter path based on patient-specific anatomy, reducing complications such as vascular injury or cardiac perforation, and AI can monitor ICE images continuously for early signs of complications, such as pericardial effusion or device malposition, allowing for immediate corrective action and improving procedural safety as has been seen in other areas of echocardiography [62,63].

## Data Availability

The data presented in this study are available on a reasonable request from the corresponding author.
